# Cost differences between digital tomosynthesis and standard digital mammography in a breast cancer screening programme: results from the To-Be trial in Norway

**DOI:** 10.1007/s10198-019-01094-7

**Published:** 2019-08-09

**Authors:** Tron Anders Moger, Jayson O. Swanson, Åsne Sørlien Holen, Berit Hanestad, Solveig Hofvind

**Affiliations:** 1 Department of Health Management and Health Economics, Institute of Health and Society, University of Oslo, Oslo, Norway; 2 Cancer Registry of Norway, Oslo, Norway; 3 Haukeland University Hospital, Bergen, Norway; 4 Oslo Metropolitan University, Oslo, Norway

**Keywords:** Screening, Costs, Breast cancer, Mammography, Tomosynthesis, I180, H510

## Abstract

**Background:**

Several studies in Europe and the US have shown promising results favouring digital breast tomosynthesis compared to standard digital mammography (DM). However, the costs of implementing the technology in screening programmes are not yet known.

**Methods:**

A randomised controlled trial comparing the results from digital breast tomosynthesis including synthetic mammograms (DBT) vs. DM was performed in Bergen during 2016 and 2017 as a part of BreastScreen Norway. The trial included 29,453 women and allowed for a detailed comparison of procedure use and screening, recall and treatment costs estimated at the individual level.

**Results:**

The increased cost of equipment, examination and reading time with DBT vs. DM was €8.5 per screened woman (95% CI 8.4−8.6). Costs of DBT remained significantly higher after adding recall assessment costs, €6.2 (95% CI 4.6−7.9). Substantial reductions in either examination and reading times, price of DBT equipment or price of IT storage and connectivity did not change the conclusion. Adding treatment costs resulted in too wide confidence intervals to draw definitive conclusions (additional costs of tomosynthesis €9.8, 95% CI –56 to 74). Performing biopsy at recall, radiation therapy and chemotherapy was significantly more frequent among women screened with DBT.

**Conclusion:**

The results showed lower incremental costs of DBT vs. DM, compared to what is found in previous cost analyses of DBT and DM. However, the incremental costs were still higher for DBT compared with DM after including recall costs. Further studies with long-term treatment data are needed to understand the complete costs of implementing DBT in screening.

## Introduction

Several non-randomised studies in both Europe and the US have shown promising results in favour of using digital breast tomosynthesis plus digital mammography (DBT + DM) as a screening tool, compared to standard digital mammography (DM) [1–8]. Digital breast tomosynthesis is an imaging technology providing three-dimensional reconstructions of the breast from a series of low-dose mammographic exposures, over a limited angular range. In contrast, DM only provides two-dimensional images, which could impede breast cancer detectability in some cases. The results of studies include higher cancer detection rates, as well as lower rates of false positives and recall assessments. Some studies from the US suggest DBT + DM as cost-effective compared with DM alone [9–12]. However, longer reading times [13–15], higher investment costs for IT storage and connectivity and possible overdiagnosis [16] make DBT + DM more costly. This has led to calls for more evidence before the technology can be implemented as a screening tool for breast cancer [16–19]. There are also concerns about increased radiation doses when using DBT + DM. Replacing the DM with synthetic 2D mammographic images (SM) reconstructed from the DBT projection data could reduce the challenge related to radiation dose [20].

In summary, although the use of DBT is increasing, there is a lack of knowledge on the advantages and disadvantages of the technology in several areas, including costs. On the one hand, DBT will have higher costs than DM due to the additional investment required and expected longer times used for screen readings. On the other hand, possibly lower recall rates, less extensive diagnostic workup and treatment due to better or earlier detection as indicated in some of the aforementioned literature will reduce the cost difference per woman screened. The cost difference may even be negative after taking into account all of the procedures until the end of treatment. Comparing procedures used at recall assessment and treatment following screening with DBT vs. DM, and any resulting cost differences, is important both for evaluating the potential benefits of the technology and for future cost-effective analyses.

The Cancer Registry of Norway is responsible for the national screening programme, BreastScreen Norway, which offer biennial screening for all women aged 50−69 years [21]. The programme started in four counties in 1996 and gradually expanded to become nationwide in 2004. A randomised controlled trial started in January 2016 at Haukeland University Hospital in Bergen (the To-Be 1 trial [22]) to compare the screening methods. All of the women participating in BreastScreen Norway at Haukeland were randomised into two trial arms, either DM or DBT including SM (hereafter referred to as DBT). By the end of the inclusion period in January 2018, the study included 29,453 women. Follow-up will continue until January 2020 to identify interval breast cancers and to compare cancer detection rates for the women in the next screening round. The randomised design and detailed information collected create a complete and unique data set for comparing costs in the two arms in the trial, as the additional costs of DBT at screening and all of the subsequent diagnostic and treatment procedures are registered within the study. These include costs for DBT machines, IT storage and connectivity, examination and reading times, individual diagnostic and surgical procedures and medication use. Detailed analysis of early performance measures, such as recall and detection rates, will be described in other publications.

This paper covers all of the women who participated in the trial, from screening through any breast cancer treatment. Previous studies have used incremental costs of DBT from reimbursement guidelines or selected radiology centres [9–12]. These are not necessarily representative of public mass screening, as the investment costs for DBT are split across a much larger number of women compared to using DBT only in clinical examinations. However, the design of the To-Be trial implies that a micro-costing approach can be used for estimating the incremental costs of DBT vs. DM at screening, as the factors differing between the arms (investment costs of DBT infrastructure, examination and reading times) are recorded. Reimbursement tariffs are only needed for estimating the costs of procedures in the recall and treatment stages. In addition, the randomised design implies no biassed selection of patients to DBT vs. DM. This may have influenced earlier trials with respect to recall rates, diagnostic follow-up and treatment, and thus costs. We start by comparing procedures used at recall assessment and treatment in the two trial arms. Any difference here could indicate better or earlier detection when using DBT vs. DM. Then the incremental cost of DBT vs. DM per screened woman is analysed by adding costs stepwise: first considering the screening stage, then adding costs of the recall assessment stage and finally including costs in the treatment stage. As previously mentioned, the initial higher costs of screening in the DBT arm may be reduced or even disappear after considering the costs of subsequent diagnostic and treatment procedures.

## Methods

**Fig. 1:**
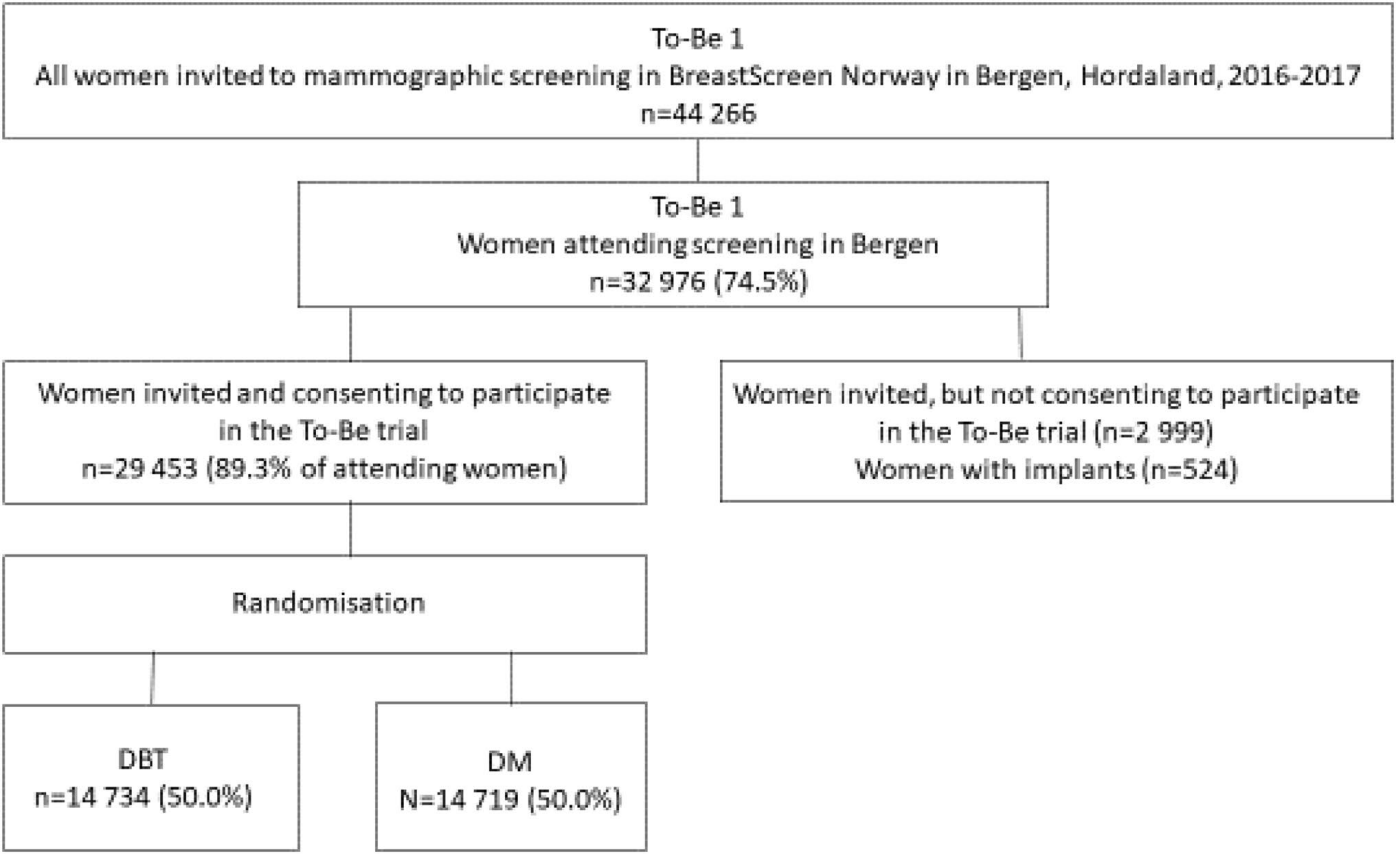
Flow chart of the recruitment process used in the To-Be1 trial. *DBT* digital breast tomosynthesis and synthetic mammography; *DM* digital mammography

The To-Be 1 trial was approved by the Regional Committees for Medical and Health Research Ethics in Norway, and registered at ClinicalTrials.gov (NCT02835625). A flow chart of the recruitment process is shown in Fig. 1. All of the women participating in the To-Be trial between January 1, 2016 and December 31, 2017, were included in the analysis. Two radiologists initially independently read each screening examination, and scored each breast on a scale from 1 to 5 (1: examinations negative for abnormality, 2: probably benign, 3: intermediate suspicion, 4: probably malignant and 5: high suspicion of malignancy). If either radiologist assigned a score of 2 or higher, a consensus meeting between two or more radiologists was held to determine whether to recall the woman for further assessment. Eight radiologists participated in the screen readings. Further details of the trial are given elsewhere [22].

All of the procedures performed at recall assessments were registered using national radiologic procedure codes. All of the surgery, chemotherapy and radiotherapy treatments were registered using corresponding national procedure codes, as well as the brand name of all of the medications prescribed to the patient. These data were acquired from each woman’s patient record at Haukeland University Hospital. The itemised cost and treatment regimens used for medications are summarised in Table 1. All of the medications used as a part of the treatment observed in the data are included in the table. This study considers only direct healthcare costs. We assumed that all of the women completed the treatment as recommended in the national guidelines (according to Table 1), and follow-up was until the end of treatment for any detected tumours. The confirmed end of treatment was recorded in the data. An exchange rate of €1.00 = NOK9.75^1^ was used throughout.

**Table 1:** Items used for estimating costs within 1 year, including medication regimens

Cost component	Source
*Screening*	
Hourly wage of radiologists and radiographers: €89 and €39	Haukeland University Hospital
Additional cost of tomosynthesis-equipped mammograph (GE SenoClaire 3D Breast Tomosynthesis™): €41,000 over 10 years using a 4% interest rate, annual cost €4900	General Electric
Additional cost of DBT vs. DM w.r.t. storage and connectivity: increased speed: €7690 per year increased storage: €23,000 per year	Project budget
*Radiology*	
Reimbursement tariff category for procedure and out-of-pocket fees	NDH [23, 24]
*In-hospital treatment*	
Procedure code and associated DRG weight, price per DRG €4385 One meeting for planning of treatment and 15 treatments assumed for radiation therapy	NDH [23, 25]
*Medication*	
Chemotherapy based on 1.8 m^2^ surface area as average: 150 mg epirubicin and 1000 mg cyclophosphamide four rounds + Neulasta 6 mg four rounds docetaxel and Taxotere 175 mg four rounds + Neulasta 6 mg four rounds paclitaxel 140 mg 12 rounds	NDH [26]
Endocrine treatment: aromatase inhibitors 12 months’ use of daily tablets + Calcigran forte 1000 mg daily + one bone scan tamoxifen 20 mg daily goserelin and Zoladex: 3.6 mg every 4 weeks, 13 rounds + one bone scan	NDH [26]
Immune therapy: Herceptin and trastuzumab 600 mg every 3 weeks, 17 rounds + echo scan four times Perjeta first treatment 840 mg intravenously, subsequent treatments 420 mg intravenously four rounds + echo scan four times	NDH [26]
Other medication: Zometa two rounds a year, dose 4 mg	NDH [26]

*DBT* digital breast tomosynthesis and synthetic mammography; *DM* digital mammography; *NDH* the Norwegian Directorate of Health; *DRG* diagnosis-related group

### Cost estimation

For the screening examinations, we estimate the incremental cost of DBT vs. DM considering factors that differ between the two. We did not aim to estimate the total cost of DBT or DM. Thus, we assumed that costs not pertaining to examination and reading times, mammograph price, and IT storage and connectivity were equal for DM and DBT. The approach implied that only factors recorded within the trial were necessary in the screening cost estimation, as detailed in the following. A pilot study conducted within the project (approximately 500 women in each arm [22]) found an average additional examination time in the laboratory of 1 min in the DBT arm. On average, 2.5 radiographers were observed to be present at the examination, which is according to normal procedures. Multiplying the time by the number of staff and the hourly wage for radiographers yielded an estimate of the incremental cost of DBT pertaining to extra examination time, applied to all of the women in the DBT arm. For the screen reading and consensus, the time used per radiologist was registered in seconds for each woman in the trial. Hence, the product of the time used and the hourly wage for radiologists yielded the estimates of cost pertaining to reading times for women in both arms. Hourly wages for radiographers and radiologists for 2017 were acquired from Haukeland University Hospital. Investment costs for the DBT were acquired from the project budget (IT storage and connectivity requirements) and General Electric (additional cost of tomosynthesis-equipped mammographs), see Table 1. These were divided equally across all of the women in the DBT arm. Two digital mammographs equipped for DBT were used in this study (General Electric SenoClaire 3D Breast Tomosynthesis™). The additional annual cost was estimated assuming a life expectancy of 10 years, and constant depreciation with a 4% capital interest rate. In this trial, the requirements of other infrastructure (for example, office space) were equal for both screening methods.

For recall assessments, costs related to radiology and subsequent workup were based on mapping procedure codes to associated reimbursement tariffs [23] in the national regulations financing public outpatient radiology services [24]. These tariffs plus patients’ out-of-pocket payments (which are €25 for imaging and €35 for intervention procedures such as biopsies) cover 40% of the resources needed for the procedure [24]; thus, the total cost of a procedure can be estimated by dividing this sum by 0.4. For in-hospital care, costs were based on mapping procedure codes [23] to associated cost weights for the corresponding diagnosis-related group (DRG) [25]. In Norway, all hospital episodes are assigned a DRG code based on the diagnosis, age, sex and procedures, resulting in a total of approximately 900 codes. For breast cancer, there are specific DRGs for radiation therapy, and different types of mastectomy, breast reconstruction among other procedures (some are listed in Table 2). The Norwegian Directorate of Health estimates the average cost for all DRG codes based on cost accounts from a sample of hospitals [25], and assign a cost weight to each code, reflecting the intensity of the treatment relative to the average patient. The cost weights depend on whether the procedure is inpatient or outpatient, the former being identified in the data from lengths of stay longer than 1 day. The cost weight multiplied by the fixed tariff per DRG point (€4385, Table 1) should cover 50% of the resource use of the procedure [25]; thus, the total cost of a procedure can be estimated by dividing this product by 0.5. DRG weights as well as DRG and radiology reimbursement tariffs for 2017 were used. The individual procedure costs were added for patients who underwent several procedures to obtain the total costs.

**Table 2:** Descriptive statistics at different stages of follow-up, where *n* denotes the number of women observed in each stage (that is, a subset of the previous stage)

Variable	DBT	DM	*P* value
Screening stage	(*n* = 14,734)	(*n* = 14,719)	
Age (SD)	59.5 (5.8)	59.5 (5.8)	0.52
Total time, screen reading (s) (SD)	132 (91)	79 (94)	< 0.001
Consensus rate (%)	6.6%	7.6%	< 0.001
Time used at consensus (s) (SD)	170 (106)	124 (124)	< 0.001
Incremental screening costs per woman, DBT (SD)	€8.5 (5.2)		< 0.001
Recall assessment stage	(*n* = 495)	(*n* = 625)	
Use of additional imaging—DM	81.4%	82.2%	0.72
Use of additional imaging—DBT	11.5%	23.2%	< 0.001
Use of ultrasound	96.4%	97.9%	0.12
Biopsy performed	52.3%	43.7%	< 0.01
Imaging cost (SD)	€50 (90)	€60 (100)	0.13
Biopsy cost (SD)	€240 (290)	€210 (260)	0.10
Total recall costs (SD)	€290 (260)	€270 (230)	0.37
Treatment stage	(*n* = 272)	(*n* = 288)	
Cancer type: benign tumour	62.1%	67.0%	0.22
Ductal carcinoma in situ	5.9%	6.2%	
Locally advanced	1.8%	3.5%	
Invasive	30.2%	23.3%	
Diagnostic biopsy	28.7%	24.7%	0.28
Any surgery	44.9%	39.6%	0.21
Wedge resection of breast	39.7%	34.7%	0.22
Excision of axillary lymph node	28.3%	23.6%	0.21
Subcutaneous mastectomy	0.4%	1.0%	0.34
Total mastectomy	5.5%	4.5%	0.59
Breast reconstruction with prosthesis	0.7%	1.7%	0.29
Total hospital length of stay (SD)	3.7 (10.1)	2.4 (3.4)	0.42
Radiation therapy	33.1%	25.4%	0.04
Chemotherapy	15.8%	9.4%	0.02
Endocrine treatment	22.1%	18.1%	0.24
Immunotherapy	2.9%	2.8%	0.91
Surgery cost (SD)	€6500 (9300)	€6400 (11,700)	0.25
Radiation therapy cost (SD)	€2000 (2800)	€1500 (2600)	0.04
Chemotherapy cost (SD)	€1300 (3500)	€700 (2500)	0.02
Medication cost (SD)	€900 (4500)	€800 (4100)	0.10
Total treatment costs (SD)	€10,700 (15,600)	€9400 (16,000)	0.16

Treatment stage includes both benign and malignant tumours. Costs are given per woman in the respective stage

*DBT* digital breast tomosynthesis and synthetic mammography; *DM* digital mammography

The purchase price of medications for 2017 was obtained from the Norwegian Medicine Agency, assuming standard treatment regimens and dosages (see Table 1) for all individuals based on the national guidelines [26]. The average height and weight of women attending screening in Hordaland (166.2 cm and 70.9 kg) were used to calculate the surface area for chemotherapy treatment [27].

### Statistical methods

Descriptive statistics for the stages: screening, recall assessment and treatment are given as means and standard deviations (SD) for continuous variables and percentages for categorical variables. For continuous variables, tests for differences between the trial arms were performed using *t* tests for age and reading times, while Mann−Whitney tests were used for cost variables due to high skewness. For categorical variables such as indicators of individual diagnostic and treatment procedures, Chi-square tests were applied. Due to space considerations, we do not show descriptive statistics for every individual procedure recorded in the data. For instance, MRI was prevalent only in supplementary and control examinations related to treatment, not in the initial recall assessments, and the number of examinations is not shown in the table.

The cost difference per screened woman was analysed in three steps: screening components only, then adding recall assessment costs and finally adding treatment costs. The screening costs varied significantly across the nine radiologists within the trial, and some performed more screen readings in one trial arm relative to the other. This may influence the estimated cost difference between the trial arms. Thus, we adjusted for radiologist fixed effects by estimating the marginal differences from gamma-GLM models with log-link, where indicator variables for the trial arm, radiologists, and interactions between these were included as covariates. Confidence intervals for the estimated cost difference were calculated for inference, using the percentile method and 10,000 bootstrap replications of the individual level data including screening, diagnostic and treatment procedures, and costs.

To study the sensitivity of the estimates and confidence intervals to women with very high costs, we removed those with total costs above €40,000 (all of whom had tumours). Furthermore, the factors presently leading to a higher cost of DBT at screening are likely reduced over time. This makes it useful to illustrate how reductions in some of these costs impact the results. Prices of IT storage and connectivity have decreased rapidly in the past, with the price of 1 GB storage halved every 1−2 years for several decades (see e.g. Fig 1 in [28]). The price of connectivity per MB/s dropped by 90% in the U.S. from 2006 to 2016 [29]. Reduced price of storage and connectivity over time could be counteracted by increased requirements of later generation machines with DBT. We studied the impact of a 50% reduction in costs of IT storage and connectivity speed. Similarly, it is possible that the increased interest in DBT and competition between manufacturers allows for a reduction in DBT purchase costs compared with DM in a few years. For example, a cost analysis of DM compared with analogue mammography performed by the UK National Health Service in 2002 [30] used a purchase price of £225,000 for a DM machine (~ €250,000, not adjusted for inflation). The price for a DM machine in the To-Be trial was €160,000, and has been stable over the last few years. A similar development in the purchase price of a machine with DBT would imply that the price difference to a DM machine decreases substantially over time. We studied the impact of a 30% decrease in the additional price over a DM machine, or from €4900 per year (Table 1) to €3400 per year. Any additional time used for the DBT examinations compared with DM is likely to decrease due to the improvements in the design of equipment and radiographers’ experience. Preliminary results from a follow-up study to To-Be 1, where the women are screened with the newer GE Senographe Pristina machine, indicate significantly lower additional examination times for DBT vs. DM than the 1 min observed in the current study. Also, previous results from the first year of the trial [22] indicated longer additional reading times in the DBT arm than were observed in other studies. Thus, we examined the impact of no additional examination time combined with a 20% decrease in the DBT total radiologist reading time. The data were analysed using Stata version 14.2, and a significance level of 5% was applied throughout.

## Results

Descriptive results are shown in Table 2. Age was similar in both trial arms. Longer reading times in the DBT arm were apparent at both screening and consensus. The consensus rate was significantly lower in the DBT arm. The unadjusted incremental screening cost for DBT was estimated at €8.5, of which 16% was due to the additional cost of the DBT equipment, 37% to storage requirements, 12% to connectivity, 19% to additional examination time and 16% to additional reading times.

Recall assessment included additional imaging and ultrasound in both arms. DBT was used significantly more frequently in the DM than the DBT arm (Table 2). In addition, more women received biopsies in the DBT vs. the DM arm. Mainly due to increased biopsy use, the point estimate for the average cost per recall was somewhat higher for DBT than DM. However, due to the large variation in the costs at this stage, the difference was not significant.

The treatment stage included both malignant and benign tumours, as 15% of benign tumours also received additional procedures after the recall assessment (for example, draining of cysts and surgical procedures). The use of most procedures did not differ between the two arms, except for more women receiving radiation therapy and chemotherapy in the DBT arm. The statistical power to detect the differences in the relative procedure frequency between the trial arms in the treatment stage is not exceptional, as the sample size is quite small here. Differences between arms in procedure frequency needed to be at least 5% points for relative frequencies below 20%, and at least 12% points for relative frequencies around 50% to reach significance. Although the substantial cost variation in the treatment stage makes it difficult to draw conclusions, the average costs of medication and surgery per treated woman were similar in the trial arms, while costs for radiation and chemotherapy, and thus the average total costs per treated woman, were higher in the DBT arm. Surgery accounted for more than 60% of the total treatment costs in both arms.

Table 3 shows the cost differences between the trial arms per screened woman. As the empirical results demonstrate, adjustment for radiologist effects did not have an impact. The incremental screening cost in Table 3 is €8.5, the same as the unadjusted estimate in Table 2. Costs in the DBT arm were significantly higher also after adding recall costs, at €6.2. Comparing these amounts imply that recall costs were on average €2.3 lower per screened woman in the DBT arm. Similarly, treatment costs were €3.6 higher per woman in the DBT arm. Even with more than 14,000 women included in each arm, the confidence intervals for the cost difference per screened woman between the trial arms were wide after adding treatment costs. The impact of the variation in treatment costs is further indicated by the fact that removing patients with costs above €40,000 (*n* = 30) resulted in a fairly large change in the point estimate for the cost difference. The sensitivity analyses showed that assuming equal examination time and a 20% reduction in the total reading time had the greatest impact on the results. However, using the confidence intervals for inference did not alter any conclusions.

**Table 3:** Cost differences per screened woman when adding costs at different stages, adjusted for radiologist effects

	Difference in costs: DBT to DM (Euro (€), 95% CI)			
	Empirical results	50% reduction in storage and connectivity costs	30% reduction in additional price of tomo-equipped mammograph	No additional examination time, 20% reduction in total reading time DBT
Incremental screening costs	€8.5 (8.4 to 8.6)	€6.4 (6.3 to 6.5)	€8.1 (8.0 to 8.2)	€6.1 (6.0 to 6.2)
Adding recall costs	€6.2 (4.6 to 7.9)	€4.1 (2.4 to 5.8)	€5.8 (4.1 to 7.5)	€3.8 (2.1 to 5.5)
Adding treatment costs	€9.8 (–56 to 74)	€7.8 (–60 to 74)	€9.4 (–59 to 73)	€7.5 (–60 to 74)
Removing 30 patients with costs > €40,000	€26.3 (–17 to 70)	€24.3 (–18 to 67)	€25.9 (–17 to 69)	€24.0 (–20 to 67)

*DBT* digital breast tomosynthesis and synthetic mammography; *DM* digital mammography; 95% *CI* 95% bootstrap percentile confidence interval

## Discussion

This study found that the incremental cost of screening with DBT vs. DM was less than €10 per woman in the study. Despite the modest incremental screening cost, costs were still significantly higher in the DBT arm after including costs related to recall assessment. This is mostly due to the recall rate not being sufficiently lower in the DBT arm of the study. However, because recall rates in Norway are generally low, and much lower than in the US [31, 32], a substantial reduction is not expected. In the DBT arm, biopsy was more frequent at recall assessment, while radiation therapy and chemotherapy were more frequent at treatment. This was expected due to a higher proportion of benign tumours in the DM arm, and characteristics of the invasive tumours detected in two arms in the To-Be trial. The latter will be discussed in a clinical companion paper. The limited number of women in the treatment stage reduces power in detecting differences in procedure use, for instance, related to detecting more cases or earlier detection in the DBT arm. Still, the findings do not indicate substantially less procedure use at recall or less intensive treatment of detected tumours when screening with DBT compared with DM.

The additional analyses showed that fairly large reductions in the incremental cost of DBT machines, storage, connectivity speed and reading times did not reduce costs in the DBT arm sufficiently to obtain a non-significant difference to DM after including recall assessment costs. The reductions are assumed to reflect possible cost developments in the near future; for instance, a reduction in reading times by 20% on average in the DBT arm corresponds to a reduction from 132 to 106 s for the screening examination, and a difference to DM similar to previous studies [1, 13–15]. The analyses illustrate that the reductions in DBT screening costs are needed to reach the cost level of DM, after all women have gone through screening, possible recall assessment and tumour treatment. However, to reach a borderline non-significant incremental cost of DBT after the recall assessment, one would need, for example, a combination of 50% reduction in storage and connectivity costs, equal examination time and a 20% reduction in reading times. The incremental cost of DBT per screened woman would then be €1.7 (95% CI 0.0−3.4).

The micro-costing approach adopted here indicated a much lower incremental cost of DBT compared to DM than applied in previous economic evaluations of DBT. Although not directly comparable as they use DBT + DM instead of DBT in the intervention group, the studies from the US [9–12] applied incremental costs of $35−50 (~ €29−41) for DBT + DM compared to DM alone. Also in contrast to the incremental cost estimated in this paper, the reimbursement tariffs for 2017 in the Norwegian radiology codes price DBT €78 higher per examination compared to DM. However, as noted in the “Introduction”, the tariff is based on using DBT for clinical examinations, which may not be representative when using DBT for a vast number of examinations in public mass screening. The machines used in the trial ran at full capacity throughout the study period, and 10−12 women were invited each hour during daytime, 5 days a week. To some extent, the incremental cost estimate from the To-Be trial could depend on the number of women screened during a year, as the price of the equipment and infrastructure related to IT storage and connectivity may not decrease in proportion to the number of women screened. Bergen is the second largest city in Norway, with a municipal population of approximately 280,000. If used in a sparsely populated area, the cost might be higher.

To illustrate the cost implications if implementing DBT in BreastScreen Norway, consider the following. In 2018, approximately 225,000 women were expected to be screened with DM, detecting around 1250 tumours in the screening [33]. Based on the incremental cost estimate and assuming the same incidence and detection rate for DBT as for DM, the additional cost of screening these women with DBT instead of DM in 2018 would be approximately €1.9 million. For direct healthcare costs, the savings per cancer detected would need to be larger than €1500 for DBT to be less expensive than DM, pertaining to a combination of fewer recall assessments, cost savings from fewer diagnostic workups and earlier detection in the recall and treatment stages. The results in the paper do not indicate that savings of that magnitude are likely. On the other hand, €1.9 million in additional screening costs per year is not a substantial amount. However, screening all of the women with DBT in Norway has some additional logistical challenges, due to the sparse populations in many municipalities and vast distances. Approximately, 20% are invited to screening at mobile units. Challenges could arise concerning connectivity and associated costs when using these, as the storage needed for DBT is much larger than for DM. According to a sample study within the To-Be trial, the size of files from a DBT examination varied between 500 and 3000 Mb, compared to 60−80 Mb for DM [22]. In conclusion, there are several considerations that have to be taken into account before implementing DBT in nationwide screening.

It is apparent that adding costs in the treatment stage greatly increased the uncertainty of the cost difference between the trial arms. This applies even though there were few cases in the treatment stage, and more than 14,000 patients included in each trial arm. Still, the sample size of the To-Be 1 trial is twice as large as several previously published clinical studies [1–3, 5]. The impact of variation in treatment costs is rarely considered in published economic evaluations of DBT. Typically, only point estimates are presented [10], or sensitivity analyses focusing on variations in the DBT + DM recall rate [9, 11], sensitivity, specificity, or additional screening cost [12].

In summary, the To-Be trial allows for a more detailed cost analysis of DBT, compared to the cost analyses seen in other economic evaluations. Even though this results in lower incremental screening cost than used in the previous studies, DBT is still significantly more expensive after adding the costs of recall assessment. It is too early to conclude with certainty whether DBT reduces the procedure and medication use in the treatment stage of breast cancer, or the impact different detection rates could have on the results. The cost effectiveness will be modelled when estimates of the interval cancer rates and cancers detected in subsequent rounds are available from the To-Be trial. This will include estimates on expected survival and costs beyond follow-up in the trial, using external data from the Cancer Registry of Norway, the literature and other sources to conduct a full analysis.

## Footnotes

1 Exchange rate in December 2018, according to the European Central Bank.
